# Supplementary material for: G-protein-coupled receptor P2Y10 facilitates chemokine-induced CD4 T cell migration through autocrine/paracrine mediators
Source: Nat Commun. 2021 Nov 23;12:6798. doi: 10.1038/s41467-021-26882-9 (PMC8611058; doi:10.1038/s41467-021-26882-9)
Supplement: Supplementary file 1 — Supplementary Information [file 41467_2021_26882_MOESM1_ESM.pdf]

**G-protein-coupled receptor P2Y10 facilitates chemokine-induced CD4 T cell migration through autocrine/paracrine mediators**

Malarvizhi Gurusamy[1], Denise Tischner[1], Jingchen Shao[1], Stephan Klatt[2], Sven Zukunft[2], Remy Bonnavion[1], Stefan Günther[3], Kai Siebenbrodt[4], Roxane-Isabelle Kestner[4], Tanja Kuhlmann[5], Ingrid Fleming[2], Stefan Offermanns[1, 6], Nina Wettschureck[1, 6, *]

**Supplementary Information**

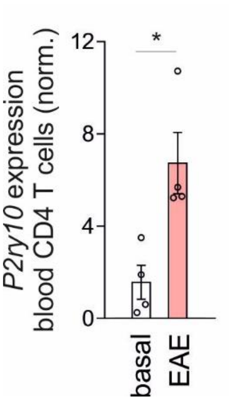


**Suppl. Fig. 1:** *P2ry10* expression in CD4 T cells isolated from peripheral blood of healthy mice (basal) or mice at peak disease of EAE (4 mice each, data are normalized to *Gapdh*). Data are means ± SEM; comparison between conditions was performed using unpaired, two-tailed Student's *t*-test; *, $p$ = 0.015.

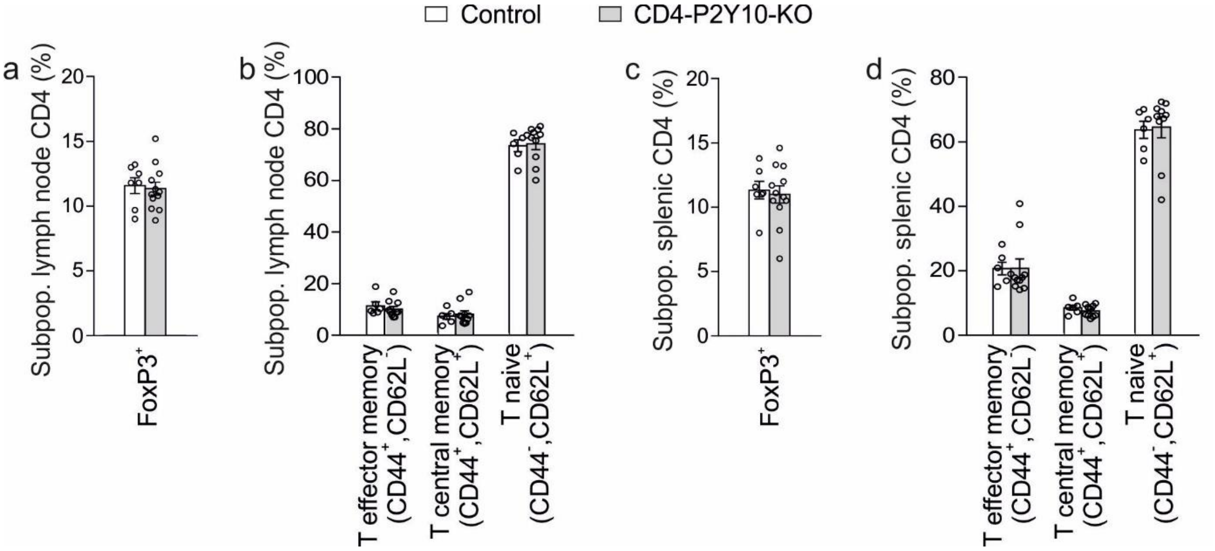


**Suppl. Fig. 2:** The percentage of FoxP3-positive regulatory T cells (a, c) or $CD44^{+}CD62L^{-}$ effector memory, $CD44^{+}CD62L^{+}$ central memory, or $CD44^{-}CD62L^{+}$ naïve T cells (b, d) was determined in CD4 T cells from inguinal lymph nodes (a,b) or spleens (c,d) (6-10 mice per group). Data are means ± SEM; comparisons between genotypes were performed using unpaired, two-tailed Student's t test.

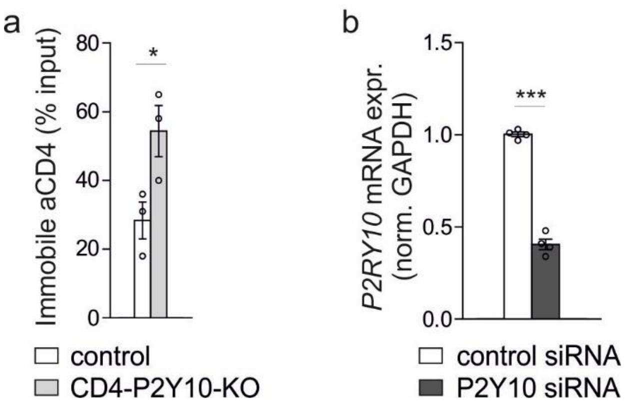


**Suppl. Fig. 3: a,** Live cell imaging analysis of migratory behavior of activated CD4 T cells in response to CCL19 stimulation (homogeneous field): Quantification of immobile versus mobile cells (cells from 3 mice per group, per experiment 44-83 cells were evaluated; *, $p = 0.047$). **b,** P2Y10 expression in siRNA-transfected human CD4 T cells was analyzed by reverse transcription polymerase chain reaction (gene expression was normalized to GAPDH and then to the average of the control group, n=4; ***, $p < 0.001$). Data are means ± SEM; comparisons between genotypes were performed using unpaired, two-tailed Student's *t*-test.

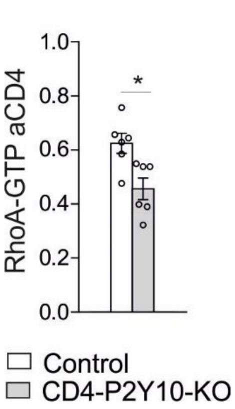


**Suppl. Fig. 4:** Basal RhoA activation in activated CD4 T cells from control and CD4-P2Y10-KOs (6 mice per group). Data are means ± SEM; comparisons between genotypes were performed using unpaired, two-tailed Student's *t*-test. *, $p = 0.011$.

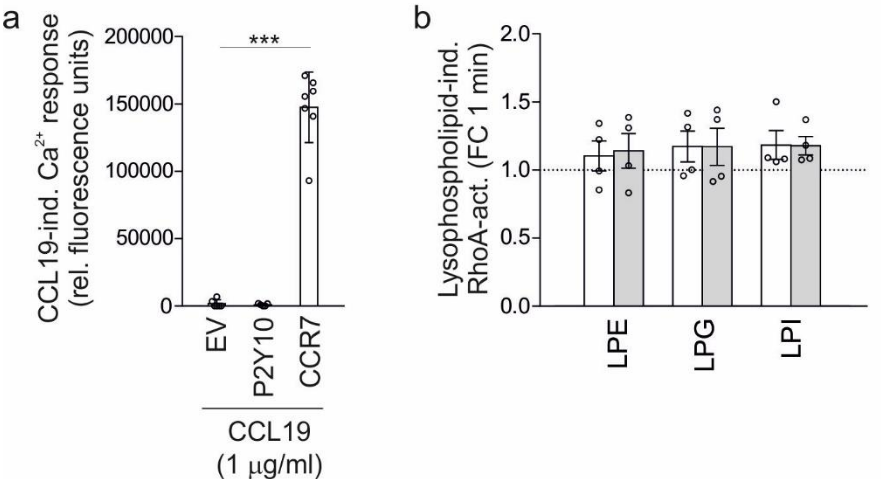


**Suppl. Fig. 5: a,** CCL19-induced calcium mobilization was determined in empty vector (EV)-, P2Y10- or CCR7-transfected COS-1 cells (n=5-7; ***, $p < 0.001$.). The CCL19 concentration used here exceeds the normally used concentration of 100 ng/ml by the factor of 10 to test whether maximal CCL19 stimulation is able to elicit a P2Y10-dependent response. **b,** RhoA activation in response to lysophosphatidylethanolamine (LPE), lysophosphatidylglycerol (LPG), or lysophosphatidylinositol (LPI) (1 μM, 1 min) in control CD4 T cells and P2Y10-deficient CD4 T cells (4 mice per group). Data are means ± SEM; comparisons between groups were performed using one-way ANOVA (a).

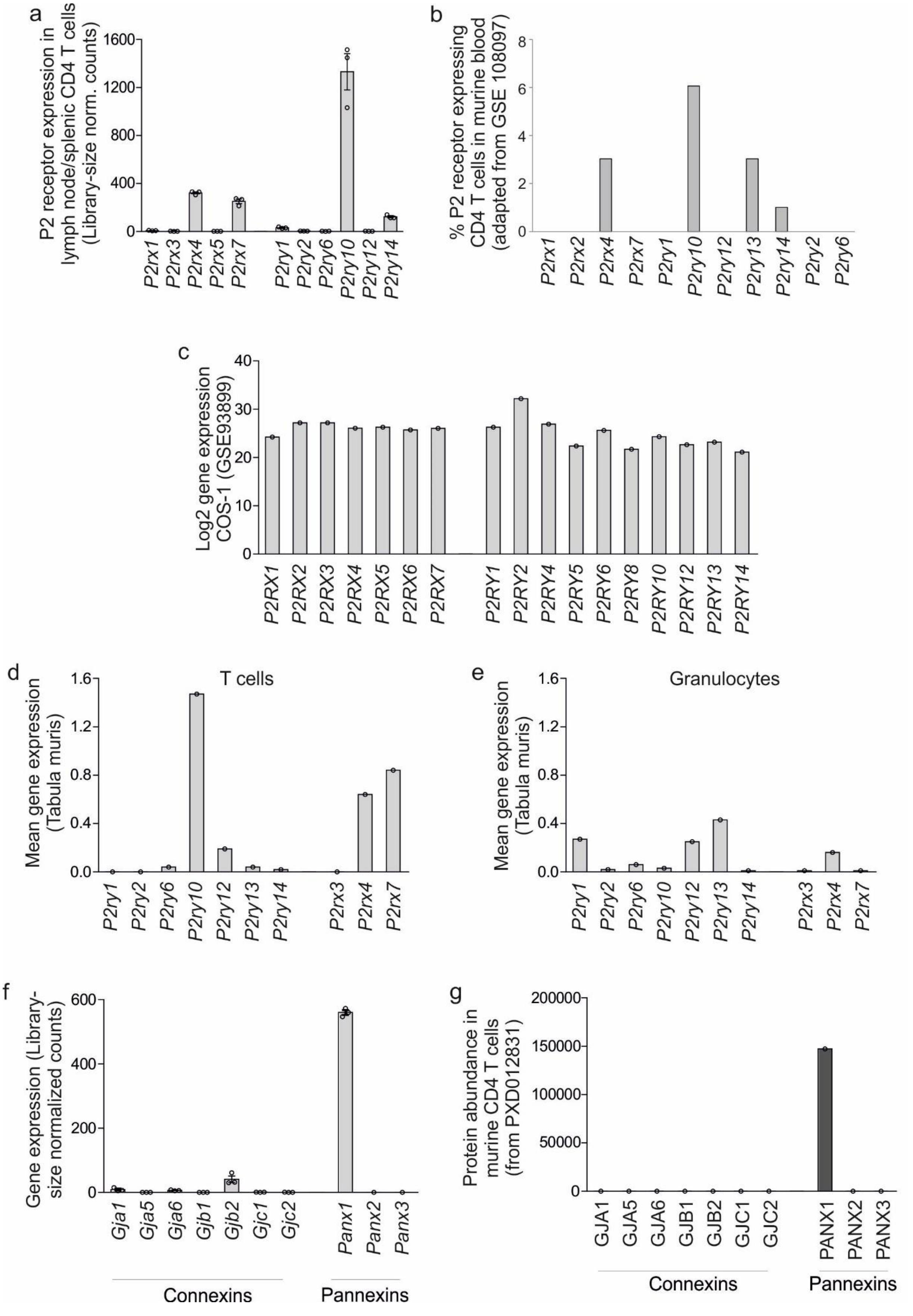

a
P2 receptor expression in lymph node/splenic CD4 T cells (Library-size norm. counts)
1600
1200
800
400
0
P2rx1 P2rx3 P2rx4 P2rx5 P2rx7 P2ry1 P2ry2 P2ry6 P2ry10 P2ry12 P2ry14
b
% P2 receptor expressing CD4 T cells in murine blood (adapted from GSE 108097)
8
6
4
2
0
P2rx1 P2rx2 P2rx4 P2rx7 P2ry1 P2ry10 P2ry12 P2ry13 P2ry14 P2ry2 P2ry6
c
Log2 gene expression COS-1 (GSE93899)
40
30
20
10
0
P2RX1 P2RX2 P2RX3 P2RX4 P2RX5 P2RX6 P2RX7 P2RY1 P2RY2 P2RY4 P2RY5 P2RY6 P2RY8 P2RY10 P2RY12 P2RY13 P2RY14
d
T cells
Mean gene expression (Tabula muris)
1.6
1.2
0.8
0.4
0.0
P2ry1 P2ry2 P2ry6 P2ry10 P2ry12 P2ry13 P2ry14 P2rx3 P2rx4 P2rx7
e
Granulocytes
Mean gene expression (Tabula muris)
1.6
1.2
0.8
0.4
0.0
P2ry1 P2ry2 P2ry6 P2ry10 P2ry12 P2ry13 P2ry14 P2rx3 P2rx4 P2rx7
f
Gene expression (Library-size normalized counts)
600
400
200
0
Gja1 Gja5 Gja6 Gjb1 Gjb2 Gjc1 Gjc2 Panx1 Panx2 Panx3
Connexins
Pannexins
g
Protein abundance in murine CD4 T cells (from PXD012831)
200000
150000
100000
50000
0
GJA1 GJA5 GJA6 GJB1 GJB2 GJC1 GJC2 PANX1 PANX2 PANX3
Connexins
Pannexins

**Suppl. Fig. 6: a,** Expression of different P2X and P2Y receptors in naïve CD4 T cells from lymph nodes and spleens was determined by mRNA sequencing (n=3). **b,** Expression of different P2X and P2Y receptors in murine peripheral blood CD4 T cells was determined by single-cell RNAseq. Data are re-analyzed from GSE 108097 [1] (100 CD4-positive and 659 CD8-positive T cells from peripheral blood of 4 healthy mice). **c,** Expression of P2X and P2Y receptors in COS-1 cells (data adapted from GSE93899). **d, e,** Expression of P2X and P2Y receptors in immature T cells (d, 60 cells) and granulocytes (e, 761 cells) from murine bone marrow (adapted from Tabula muris[2], shown is mean expression of individual cells). **f,** Expression of different connexins and pannexins in nCD4 was determined by mRNA sequencing (n=3). **g,** Abundance of different pannexins and connexins in a proteomic analysis in murine CD4 T cells (reanalyzed from PXD012831 [3]). Data are means ± SEM; no statistical testing applied.

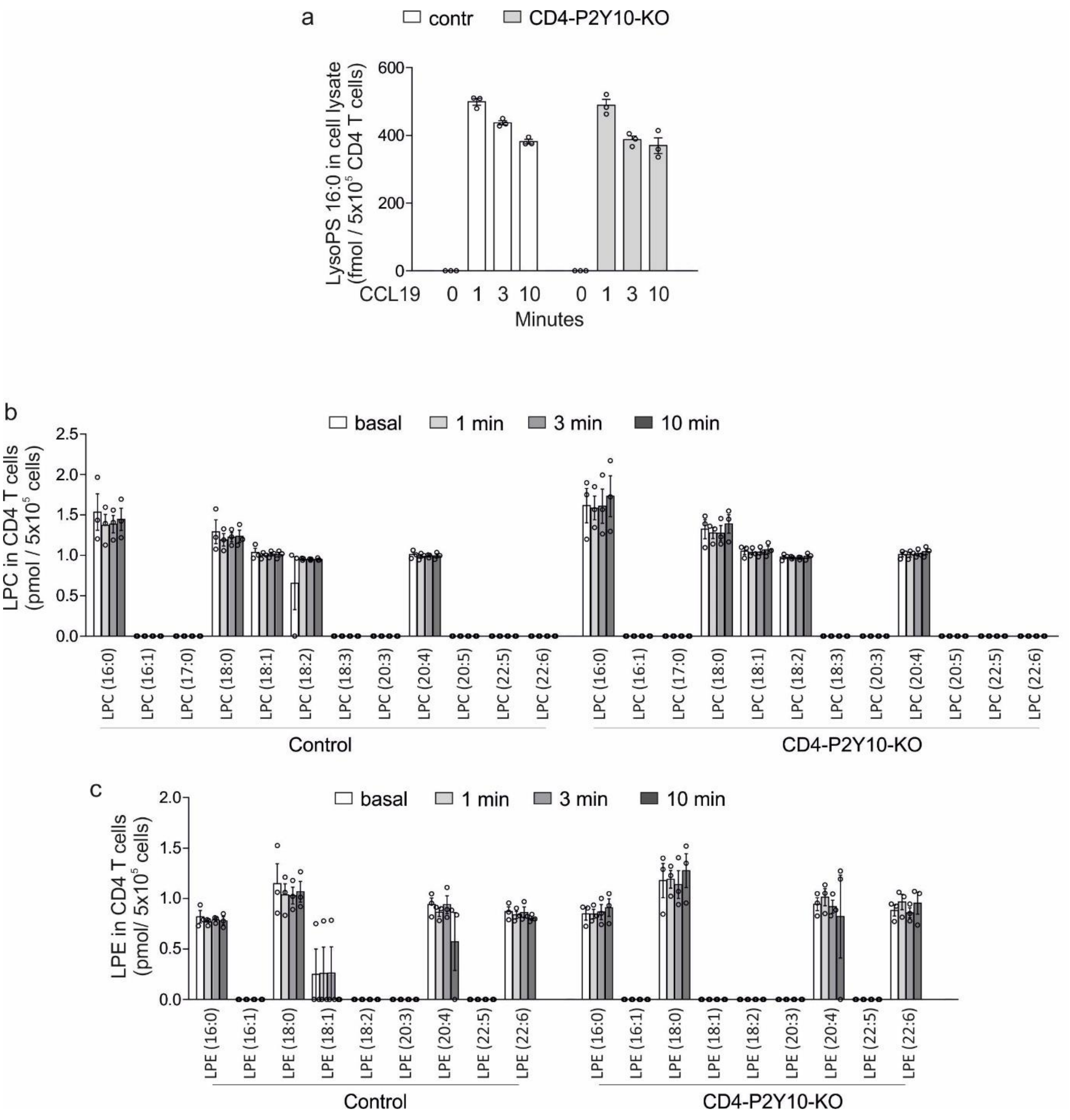


**Suppl. Fig. 7: a,** LysoPS 16:0 was detected by LC-MS/MS in in lysates of CCL19-treated CD4 T cells from control mice and CD4-P2Y10-KOs (n=3 mice per group). **b,c,** Lysophospholipid species LPC (b) and LPE (c) were determined by LC-MS/MS in lysates of 5 x 10$^5$ isolated murine CD4 T cells from control mice and CD4-P2Y10-KOs at 0, 1, 3 and 10 minutes of CCL19 (100 ng/ml) stimulation (n=3 mice per group). Data are means ± SEM; comparisons between time points were performed using two-way ANOVA with Dunnett's multiple comparisons test.

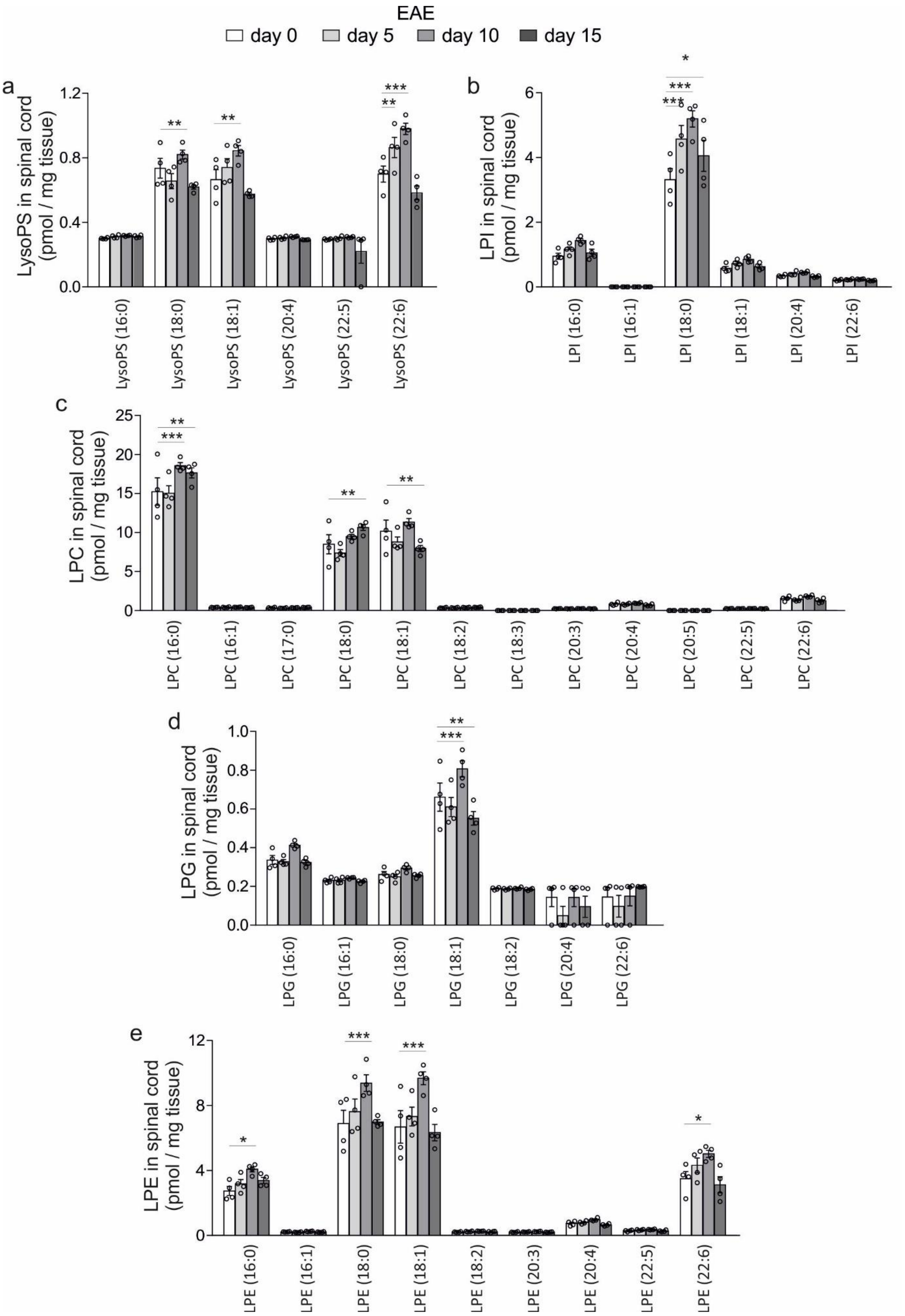
EAE
day 0
day 5
day 10
day 15
a
LysoPS in spinal cord (pmol / mg tissue)
1.2
0.8
0.4
0.0
**
**
***
**
LysoPS (16:0)
LysoPS (18:0)
LysoPS (18:1)
LysoPS (20:4)
LysoPS (22:5)
LysoPS (22:6)
b
LPI in spinal cord (pmol / mg tissue)
6
4
2
0
*
***
***
LPI (16:0)
LPI (16:1)
LPI (18:0)
LPI (18:1)
LPI (20:4)
LPI (22:6)
c
LPC in spinal cord (pmol / mg tissue)
25
20
15
10
5
0
**
***
**
**
LPC (16:0)
LPC (16:1)
LPC (17:0)
LPC (18:0)
LPC (18:1)
LPC (18:2)
LPC (18:3)
LPC (20:3)
LPC (20:4)
LPC (20:5)
LPC (22:5)
LPC (22:6)
d
LPG in spinal cord (pmol / mg tissue)
1.0
0.8
0.6
0.4
0.2
0.0
**
***
LPG (16:0)
LPG (16:1)
LPG (18:0)
LPG (18:1)
LPG (18:2)
LPG (20:4)
LPG (22:6)
e
LPE in spinal cord (pmol / mg tissue)
12
8
4
0
*
***
***
*
LPE (16:0)
LPE (16:1)
LPE (18:0)
LPE (18:1)
LPE (18:2)
LPE (20:3)
LPE (20:4)
LPE (22:5)
LPE (22:6)

**Suppl. Fig. 8:** Lysophospholipid species were determined by LC-MS/MS in spinal cords of control mice at time points 0, 5, 10, 15 after EAE induction (n=4). Data are means ± SEM; comparisons between time points were performed using two-way ANOVA with Dunnett's multiple comparisons test; *, $p < 0.05$; **, $P < 0.01$; ***, $P < 0.001$; n, number of individual mice per group.

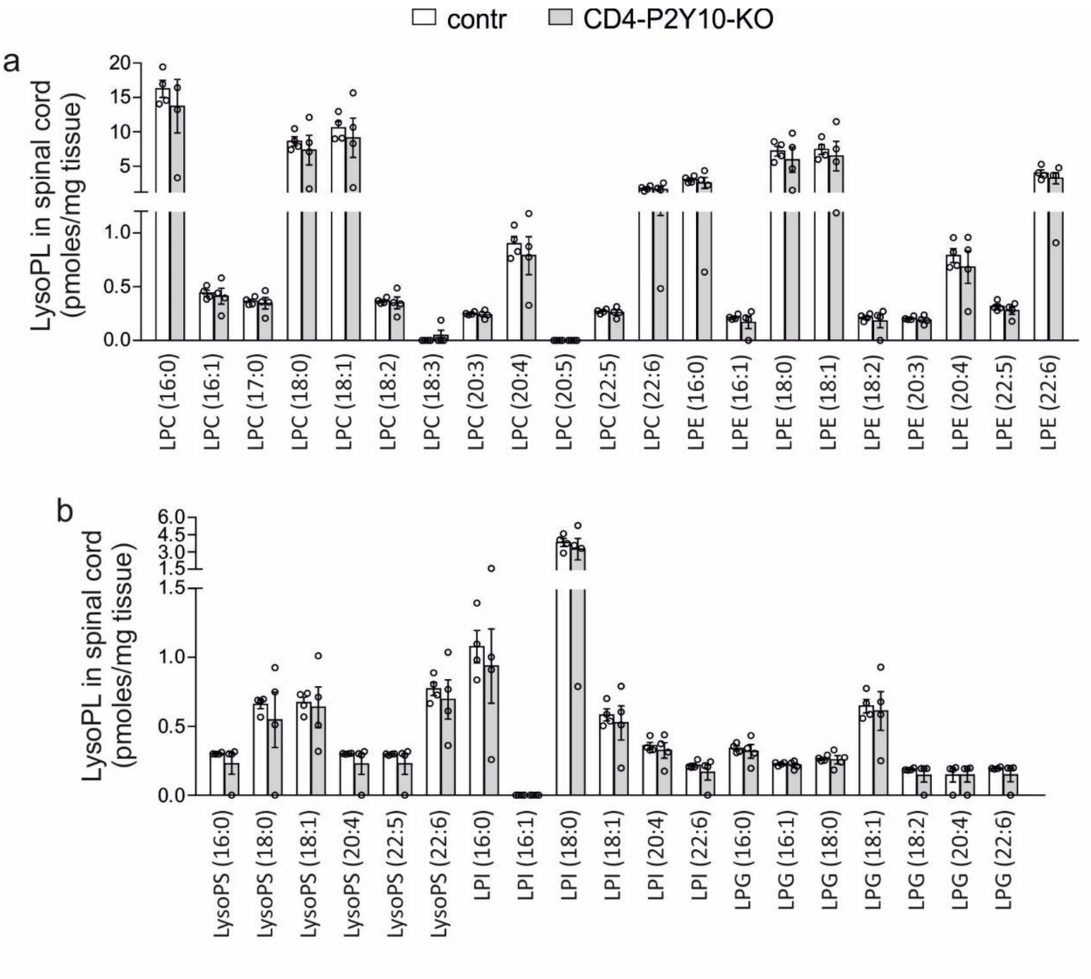


**Suppl. Fig. 9:** Lysophospholipid species were determined by LC-MS/MS in spinal cords of control and CD4-P2Y10-KOs (4 mice per group). Data are means ± SEM; comparisons between genotypes were performed using unpaired, two-tailed Student's *t*-test.

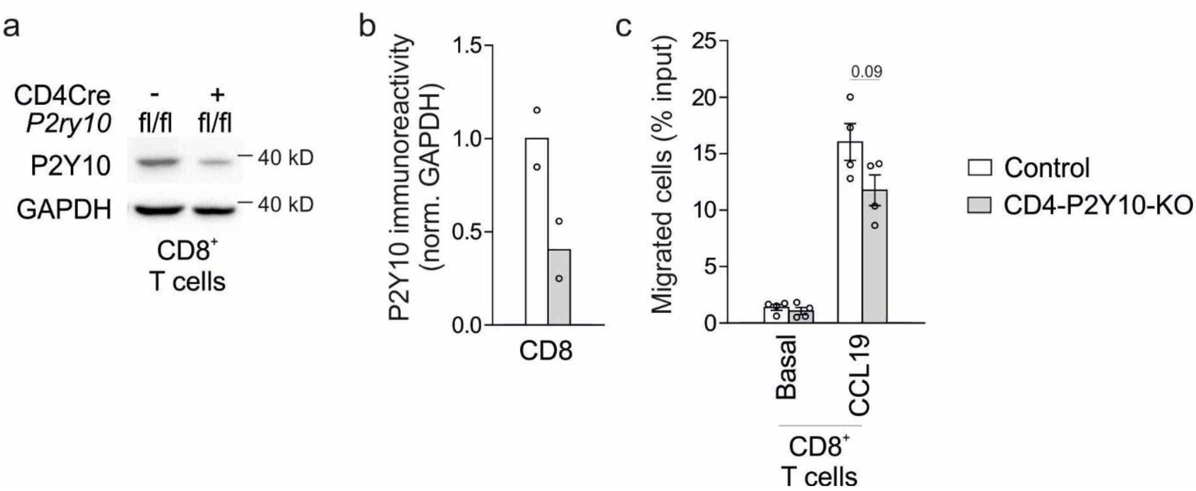


**Suppl. Fig. 10: a,b,** Reduction of P2Y10 expression in CD8 T cells isolated from control and CD4-P2Y10-KO mice as judged by immunoblotting (a, exemplary immunoblots, b, statistical evaluation). GAPDH as loading control (n=2). **c,** Transwell migration of naïve CD8 T cells under basal conditions and after addition of 100 ng/ml CCL19 to the lower well (n=4). Data are means ± SEM (in (b) only means); comparisons between genotypes were performed using unpaired, two-tailed Student's *t*-test (for c).

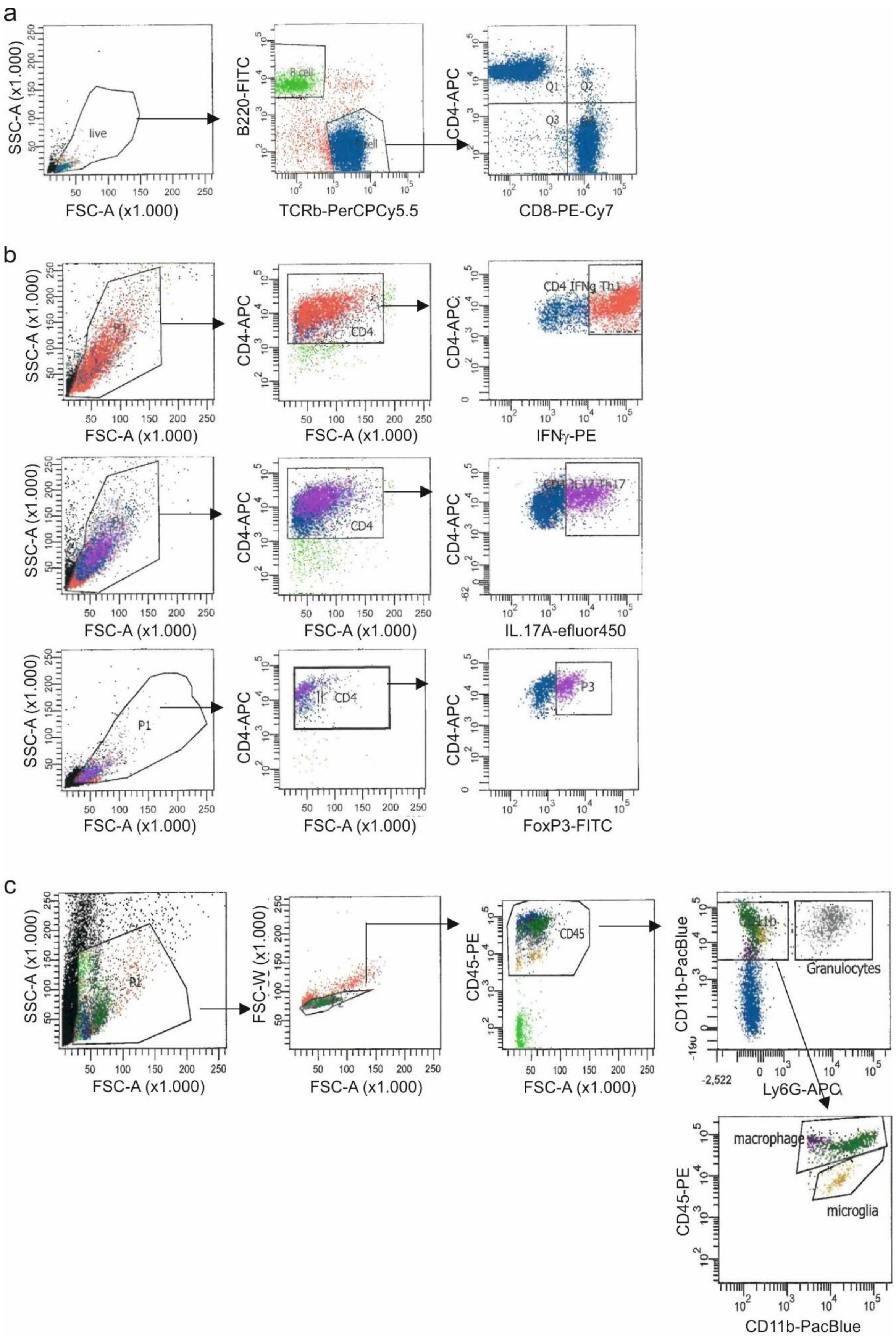
a
SSC-A (x1.000)
FSC-A (x1.000)
live
B220-FITC
B cell
TCRb-PerCPCy5.5
CD4-APC
Q1
Q2
Q3
CD8-PE-Cy7
b
P1
CD4
CD4 IFNg Th1
IFNγ-PE
IL.17A-efluor450
P3
FoxP3-FITC
c
FSC-W (x1.000)
CD45-PE
CD45
CD11b-PacBlue
Granulocytes
Ly6G-APC
macrophage
microglia

**Suppl. Fig. 11:** Gating strategies for **a,** the analysis of leukocyte populations in spleen/lymph node/thymus (pertaining to Figures 1d-h); **b,** cytokine expression in vitro-differentiated CD4 T cells (pertaining to Figures 1k); **c,** and spinal cord-infiltrating leukocytes in EAE mice (pertaining to Figure 2).

## Supplementary Tables for Gurusamy et al., 2021

| Positive Ionization Mode | | | | | | |
|---|---|---|---|---|---|---|
| Lyso-Lipid | Q1 | Q3 | Dwell Time | DP | CE | CXP |
| LPC (14:0) | 468,3 | 184,1 | 4 | 90 | 10 | 12 |
| LPC (16:0) | 496,3 | 184,1 | 4 | 90 | 38 | 12 |
| LPC (16:1) | 494,3 | 184,1 | 4 | 90 | 38 | 12 |
| LPC (17:0) | 510,4 | 184,1 | 4 | 90 | 38 | 12 |
| LPC (18:0) | 524,4 | 184,1 | 4 | 90 | 38 | 12 |
| LPC (18:1) | 522,4 | 184,1 | 4 | 90 | 38 | 12 |
| LPC (18:2) | 520,3 | 184,1 | 4 | 90 | 38 | 12 |
| LPC (18:3) | 518,3 | 184,1 | 4 | 90 | 38 | 12 |
| LPC (20:3) | 546,4 | 184,1 | 4 | 90 | 38 | 12 |
| LPC (20:4) | 544,3 | 184,1 | 4 | 90 | 38 | 12 |
| LPC (20:5) | 542,3 | 184,1 | 4 | 90 | 38 | 12 |
| LPC (22:5) | 570,4 | 184,1 | 4 | 90 | 38 | 12 |
| LPC (22:6) | 568,3 | 184,1 | 4 | 90 | 38 | 12 |
| 18:1-d7 Lyso-PC | 529,4 | 184,1 | 4 | 80 | 38 | 13 |
| LPI (14:0) | 545,33 | 285,33 | 4 | 80 | 31 | 10 |
| LPI (16:0) | 573,33 | 313,33 | 4 | 80 | 31 | 10 |
| LPI (16:1) | 571,33 | 311,33 | 4 | 80 | 31 | 10 |
| LPI (18:0) | 601,33 | 341,33 | 4 | 80 | 31 | 10 |
| LPI (18:1) | 599,33 | 339,33 | 4 | 80 | 31 | 10 |
| LPI (18:2) | 597,33 | 337,33 | 4 | 80 | 31 | 10 |
| LPI (18:3) | 595,23 | 335,23 | 4 | 80 | 31 | 10 |
| LPI (20:3) | 623,33 | 363,33 | 4 | 80 | 31 | 10 |
| LPI (20:4) | 621,33 | 361,33 | 4 | 80 | 31 | 10 |
| LPI (20:5) | 619,33 | 359,33 | 4 | 80 | 31 | 10 |
| LPI (22:5) | 647,33 | 387,33 | 4 | 80 | 31 | 10 |
| LPI (22:6) | 645,33 | 385,33 | 4 | 80 | 31 | 10 |
| LPG (14:0) | 457,2 | 285,33 | 4 | 80 | 26 | 10 |
| LPG (16:0) | 485,3 | 313,33 | 4 | 80 | 26 | 10 |
| LPG (16:1) | 483,3 | 311,33 | 4 | 80 | 26 | 10 |
| LPG (18:0) | 513,3 | 341,33 | 4 | 80 | 26 | 10 |
| LPG (18:1) | 511,3 | 339,33 | 4 | 80 | 26 | 10 |
| LPG (18:2) | 509,3 | 337,33 | 4 | 80 | 26 | 10 |
| LPG (18:3) | 507,3 | 335,23 | 4 | 80 | 26 | 10 |
| LPG (20:3) | 535,3 | 363,33 | 4 | 80 | 26 | 10 |
| LPG (20:4) | 533,3 | 361,33 | 4 | 80 | 26 | 10 |
| LPG (20:5) | 531,3 | 359,33 | 4 | 80 | 26 | 10 |
| LPG (22:5) | 559,3 | 387,33 | 4 | 80 | 26 | 10 |
| LPG (22:6) | 557,3 | 385,33 | 4 | 80 | 26 | 10 |
| LPE (14:0) | 426,2 | 285,33 | 4 | 80 | 31 | 10 |
| LPE (16:0) | 454,3 | 313,33 | 4 | 80 | 31 | 10 |
| LPE (16:1) | 452,3 | 311,33 | 4 | 80 | 31 | 10 |
| LPE (18:0) | 482,3 | 341,33 | 4 | 80 | 31 | 10 |
| LPE (18:1) | 480,3 | 339,33 | 4 | 80 | 31 | 10 |
| LPE (18:2) | 478,3 | 337,33 | 4 | 80 | 31 | 10 |
| LPE (18:3) | 476,3 | 335,23 | 4 | 80 | 31 | 10 |
| LPE (20:3) | 504,3 | 363,33 | 4 | 80 | 31 | 10 |
| LPE (20:4) | 502,3 | 361,33 | 4 | 80 | 31 | 10 |
| LPE (20:5) | 500,3 | 359,33 | 4 | 80 | 31 | 10 |
| LPE (22:5) | 528,3 | 387,33 | 4 | 80 | 31 | 10 |
| LPE (22:6) | 526,3 | 385,33 | 4 | 80 | 31 | 10 |
| LPS (14:0) | 470,2 | 185 | 5 | 80 | 10 | 22 |
| LPS (16:0) | 498,3 | 185 | 5 | 80 | 10 | 22 |
| LPS (16:1) | 496,3 | 185 | 5 | 80 | 10 | 22 |
| LPS (18:0) | 526,3 | 185 | 5 | 80 | 10 | 22 |
| LPS (18:1) | 524,3 | 185 | 5 | 80 | 10 | 22 |
| LPS (18:2) | 522,3 | 185 | 5 | 80 | 10 | 22 |
| LPS (18:3) | 520,3 | 185 | 5 | 80 | 10 | 22 |
| LPS (20:3) | 548,3 | 185 | 5 | 80 | 10 | 22 |
| LPS (20:4) | 546,3 | 185 | 5 | 80 | 10 | 22 |
| LPS (20:5) | 544,3 | 185 | 5 | 80 | 10 | 22 |
| LPS (22:5) | 572,3 | 185 | 5 | 80 | 10 | 22 |
| LPS (22:6) | 570,3 | 185 | 5 | 80 | 10 | 22 |

| Negative Ionization Mode | | | | | | |
|---|---|---|---|---|---|---|
| Lyso-Lipid | Q1 | Q3 | Dwell Time | DP | CE | CXP |
| LPA (14:0) | 381,2 | 153 | 4 | -100 | -22 | -10 |
| LPA (16:0) | 409,2 | 153 | 4 | -100 | -22 | -10 |
| LPA (16:1) | 407,2 | 153 | 4 | -100 | -22 | -10 |
| LPA (17:0) | 423,3 | 153 | 4 | -100 | -22 | -10 |
| LPA (18:0) | 437,3 | 153 | 4 | -100 | -22 | -10 |
| LPA (18:1) | 435,3 | 153 | 4 | -100 | -22 | -10 |
| LPA (18:2) | 433,2 | 153 | 4 | -100 | -22 | -10 |
| LPA (18:3) | 431,2 | 153 | 4 | -100 | -22 | -10 |
| LPA (20:3) | 459,3 | 153 | 4 | -100 | -22 | -10 |
| LPA (20:4) | 457,2 | 153 | 4 | -100 | -22 | -10 |
| LPA (20:5) | 455,2 | 153 | 4 | -100 | -22 | -10 |
| LPA (22:5) | 483,3 | 153 | 4 | -100 | -22 | -10 |
| LPA (22:6) | 481,2 | 153 | 4 | -100 | -22 | -10 |
| LPS (14:0) | 468,2 | 381,2 | 5 | -100 | -22 | -10 |
| LPS (16:0) | 496,3 | 409,2 | 5 | -100 | -22 | -10 |
| LPS (16:1) | 494,3 | 407,2 | 5 | -100 | -22 | -10 |
| LPS (18:0) | 524,3 | 437,3 | 5 | -100 | -22 | -10 |
| LPS (18:1) | 522,3 | 435,3 | 5 | -100 | -22 | -10 |
| LPS (18:2) | 520,3 | 433,2 | 5 | -100 | -22 | -10 |
| LPS (18:3) | 518,3 | 431,2 | 5 | -100 | -22 | -10 |
| LPS (20:3) | 546,3 | 459,2 | 5 | -100 | -22 | -10 |
| LPS (20:4) | 544,3 | 457,2 | 5 | -100 | -22 | -10 |
| LPS (20:5) | 542,3 | 455,2 | 5 | -100 | -22 | -10 |
| LPS (22:5) | 570,3 | 483,3 | 5 | -100 | -22 | -10 |
| LPS (22:6) | 568,3 | 481,2 | 5 | -100 | -22 | -10 |

**Supplementary Table 1:** Additional information about analyzed lipids: Q1/Q3 transitions, dwell time, declustering potential (DP), collision energy (CE) and collision cell exit potential (CXP).

| | forward | reverse |
|---|---|---|
| Murine *P2ry10* | 5-AGTCTTCGTTATCTGCTTCACT-3 | 5-GATGGAAATACAGGGTACTTTT-3 |
| Murine *Gapdh* | 5- AGACGGCCGCATCTTCTT-3 | 5- TTCACACCGACCTTCACCAT-3 |
| Human *P2RY10* | 5-TGCAGTATAAATAGCATAGACATTTGG-3 | 5-TTCATTCTTCCATAGTCACTCTGTTC-3 |
| Human *GAPDH* | 5- GCATCCTGGGCTACACTGA-3 | 5-CCAGCGTCAAAGGTGGAG-3 |
| Genotyping primers 7F/7R (left loxP) | 5-AACTGCCATCTTCACTCAGTTG-3 | 5-CAGATGCCATAGGTTTTGTTAGC-3 |
| Genotyping primers 5F/5R (right loxP) | 5-CCCCAGGGTCATGAGAGTAG-3 | 5-GTCATTAACCCCACCACCAC-3 |

**Supplementary Table 2:** Primer sequences for RT-PCR.

**Supplementary References for Gurusamy et al., 2021**

1. Han X, Wang R, Zhou Y, Fei L, Sun H, Lai S, Saadatpour A, Zhou Z, Chen H, Ye F, Huang D, Xu Y, Huang W, Jiang M, Jiang X, Mao J, Chen Y, Lu C, Xie J, Fang Q, Wang Y, Yue R, Li T, Huang H, Orkin SH, Yuan GC, Chen M and Guo G. Mapping the Mouse Cell Atlas by Microwell-Seq. *Cell*. 2018;173:1307.
2. Tabula Muris C, Overall c, Logistical c, Organ c, processing, Library p, sequencing, Computational data a, Cell type a, Writing g, Supplemental text writing g and Principal i. Single-cell transcriptomics of 20 mouse organs creates a Tabula Muris. *Nature*. 2018;562:367-372.
3. Dybas JM, O'Leary CE, Ding H, Spruce LA, Seeholzer SH and Oliver PM. Integrative proteomics reveals an increase in non-degradative ubiquitylation in activated CD4(+) T cells. *Nat Immunol*. 2019;20:747-755.
