# Supplementary material for: G-protein-coupled receptor P2Y10 facilitates chemokine-induced CD4 T cell migration through autocrine/paracrine mediators
Source: Nat Commun. 2021 Nov 23;12:6798. doi: 10.1038/s41467-021-26882-9 (PMC8611058; doi:10.1038/s41467-021-26882-9)
Supplement: Supplementary file 3 — Description of Additional Supplementary Files [file 41467_2021_26882_MOESM3_ESM.pdf]

**Description of Additional Supplementary Files**

File Name: Supplementary Movie 1
Description: Exemplary movie showing the tracking of CD4 T cells migrating towards a gradient of CCL19 (100 ng/ml CCL19) in µ-Slide Chemotaxis chambers (Ibidi). For details, see section "Live-cell imaging of directed CCL19-induced migration" in Methods.
