# Supplementary material for: G-protein-coupled receptor P2Y10 facilitates chemokine-induced CD4 T cell migration through autocrine/paracrine mediators
Source: Nat Commun. 2021 Nov 23;12:6798. doi: 10.1038/s41467-021-26882-9 (PMC8611058; doi:10.1038/s41467-021-26882-9)
Supplement: Supplementary file 5 — Reporting Summary [file 41467_2021_26882_MOESM5_ESM.pdf]

nature research

Corresponding author(s): Nina Wettschureck

Last updated by author(s): 2021-07-23 (VersA), 2021-09-22 (VersB)

Reporting Summary

Nature Research wishes to improve the reproducibility of the work that we publish. This form provides structure for consistency and transparency in reporting. For further information on Nature Research policies, see our Editorial Policies and the Editorial Policy Checklist.

Statistics

For all statistical analyses, confirm that the following items are present in the figure legend, table legend, main text, or Methods section.

n/a	Confirmed	
☐	☒	The exact sample size (*n*) for each experimental group/condition, given as a discrete number and unit of measurement
☐	☒	A statement on whether measurements were taken from distinct samples or whether the same sample was measured repeatedly
☐	☒	The statistical test(s) used AND whether they are one- or two-sided<br>*Only common tests should be described solely by name; describe more complex techniques in the Methods section.*
☐	☒	A description of all covariates tested
☐	☒	A description of any assumptions or corrections, such as tests of normality and adjustment for multiple comparisons
☐	☒	A full description of the statistical parameters including central tendency (e.g. means) or other basic estimates (e.g. regression coefficient) AND variation (e.g. standard deviation) or associated estimates of uncertainty (e.g. confidence intervals)
☐	☒	For null hypothesis testing, the test statistic (e.g. *F*, *t*, *r*) with confidence intervals, effect sizes, degrees of freedom and *P* value noted<br>*Give P values as exact values whenever suitable.*
☒	☐	For Bayesian analysis, information on the choice of priors and Markov chain Monte Carlo settings
☒	☐	For hierarchical and complex designs, identification of the appropriate level for tests and full reporting of outcomes
☒	☐	Estimates of effect sizes (e.g. Cohen's *d*, Pearson's *r*), indicating how they were calculated

Our web collection on statistics for biologists contains articles on many of the points above.

Software and code

Policy information about availability of computer code

Data collection	no software used
Data analysis	mRNA sequencing raw reads were assessed for quality, adapter content and duplication rates with FastQC (version 0.11.8) (Available online at: http://www.bioinformatics.babraham.ac.uk/projects/fastqc). Trimmomatic version 0.39 was employed to trim reads after a quality drop below a mean of Q20 in a window of 5 nucleotides 64. Only reads between 30 and 150 nucleotides were cleared for further analyses. Trimmed and filtered reads were aligned versus the Ensembl mouse genome version mm10 (GRCm38) using STAR 2.7.3a with the parameter “--outFilterMismatchNoverLmax 0.1” to increase the maximum ratio of mismatches to mapped length to 10% (Dobin et al., STAR: ultrafast universal RNA-seq aligner). The number of reads aligning to genes was counted with featureCounts 1.6.5 tool from the Subread package 65. Only reads mapping at least partially inside exons were admitted and aggregated per gene. Reads overlapping multiple genes or aligning to multiple regions were excluded. The Ensemble annotation was enriched with UniProt data (release 06.06.2014) based on Ensembl gene identifiers (Activities at the Universal Protein Resource (UniProt)). The raw count matrix was batch corrected using CountClust 66 and then normalized with DESeq2. Differentially expressed genes were identified using DESeq2 version 1.26.0 (Love et al., Moderated estimation of fold change and dispersion for RNA-Seq data with DESeq2).

For manuscripts utilizing custom algorithms or software that are central to the research but not yet described in published literature, software must be made available to editors and reviewers. We strongly encourage code deposition in a community repository (e.g. GitHub). See the Nature Research guidelines for submitting code & software for further information.

Data

Policy information about availability of data

All manuscripts must include a data availability statement. This statement should provide the following information, where applicable:

- Accession codes, unique identifiers, or web links for publicly available datasets
- A list of figures that have associated raw data
- A description of any restrictions on data availability

The accession code for mRNA sequencing data is GSE162246 and data are now accessible.

Field-specific reporting

Please select the one below that is the best fit for your research. If you are not sure, read the appropriate sections before making your selection.

☒ Life sciences ☐ Behavioural & social sciences ☐ Ecological, evolutionary & environmental sciences

For a reference copy of the document with all sections, see nature.com/documents/nr-reporting-summary-flat.pdf

Life sciences study design

All studies must disclose on these points even when the disclosure is negative.

Sample size	Sample size was determined based on previous experiments of the same type, sample sizes shown in literature, or according to D. Altman (Practical Statistics for Medical Research, 1991)
Data exclusions	Is some cases individual data from individual mice had to be excluded because re-genotyping of respective mice did not confirm the genotype (pertaining to all experiments involving genetically modified mice). In other cases data from in vitro experiments were excluded, since in vitro stimulation or differentiation failed in a certain batch of cells (as judged by FACS-based analysis of activation/differentiation markers; pertaining to all experiments involving in vitro stimulation). These exclusion criteria were preestablished.
Replication	Number of independent experiments are given in the Figure Legend.
Randomization	Samples were alllocated to their respective groups based on their genetic modification (control vs KO).
Blinding	Invesitigators were blinded to genotype during data collection and analysis.

Reporting for specific materials, systems and methods

We require information from authors about some types of materials, experimental systems and methods used in many studies. Here, indicate whether each material, system or method listed is relevant to your study. If you are not sure if a list item applies to your research, read the appropriate section before selecting a response.

Materials & experimental systems

n/a	Involved in the study
☐	☒ Antibodies
☐	☒ Eukaryotic cell lines
☒	☐ Palaeontology and archaeology
☐	☒ Animals and other organisms
☐	☒ Human research participants
☒	☐ Clinical data
☒	☐ Dual use research of concern

Methods

n/a	Involved in the study
☒	☐ ChIP-seq
☐	☒ Flow cytometry
☒	☐ MRI-based neuroimaging

Antibodies

Antibodies used	For the analysis of leukocyte populations from lymphoid organs, the following antibodies were used: rat anti-mouse CD4-FITC (clone GK1.5, #11004182,1:100 dilution eBioscience), rat anti-mouse CD8a-PE-Cy7 (clone 53-6.7, #25008182, 1:100 dilution ,eBioscience) (for thymus); rat anti-mouse CD4-APC (clone RM4-5, #17004281, 1:100 dilution, eBioscience), rat anti-mouse CD8a-PE-Cy7 (clone 53-6.7, #25008182, 1:100 dilution, eBioscience), rat anti-mouse CD45R/B220-FITC (clone RA3-6B2, #554880, Becton Dickinson). For the analysis of spinal cord-infiltrating cells we used rat anti-mouse CD45-PE (clone 30-F11, #12045182, 1:100 dilution, eBioscience), rat anti-mouse Ly6g-APC (clone 1A8, #127613, 1:100 dilution, Biolegend), rat anti-mouse CD11b-eFluor 450 (clone M1/70, #48011282, 1:100 dilution, eBioscience), rat anti-mouse Ly6c-PE-Cy7 (clone HK1.417, #128017, 1:100 dilution, Biolegend), rat anti-mouse F4/80-PerCP-Cy5.5 (clone BM8, #45480182, 1:100 dilution, eBioscience), rat anti-mouse CD62L-FITC (clone MEL14, #553150, 1:100 dilution, BD Bioscience), rat anti-mouse CD44-PE (clone IM7, #553134, 1:100 dilution, BD Bioscience). For intracellular cytokine staining in spinal cord-infiltrating cells we used ,anti IFNγ-PE (clone XMG1.2, # 554412, 1:100 dilution, BD

Bioscience), anti CD4-APC (clone RM4-5, #17004281, 1:100 dilution, eBioscience), anti-IL17-A-eFluor 450 (clone eBio17B7, #48717782, 1:100 dilution, eBioscience), and rat anti-mouse GM-CSF-PerCP-Cy5.5 (clone MP1-22E9, #505409, 1:100 dilution, Biolegend).
For the analysis of spinal cord-infiltrating Treg, the following antibodies were used: anti FoxP3-FITC (clone FJK-16s, #11577382, 1:100 dilution, Thermo Fisher Scientific), rat anti-mouse CD25-Billiant Violet 421 (clone PC61, catalogue number-BLD102033, 1:100 dilution Biolegend), anti CD4-APC (clone RM4-5, #17004281, 1:100 dilution, eBioscience), rat anti-mouse CD152/CTLA-4-PE (clone UC10-4B9, #12152282, Thermo Fischer Scientific), anti CD8a-PECy7(clone 53-6.7, #25008182, 1:100 dilution, Thermo Fischer Scientific), rat anti-mouse TCR β chain-PerCP-Cy5.5 (clone H57-597, #109227, 1:100 dilution, Biolegend).
For intracellular cytokine staining in in vitro-differentiated CD4 cells, we used CD16/CD32 (FcBlock, clone 2.4G2, #553141, 1:200 dilution, Becton Dickinson) and anti-CD4-APC for extracellular staining, following intracellular staining using rat anti-mouse FoxP3 (clone FJK-16s, #11577382, 1:100 dilution, Thermo Fisher Scientific) (for iTreg), rat anti-mouse IFNγ –PE (clone XMG1.2, #554412, 1:100 dilution, BD Bioscience) and rat anti-mouse anti-IL17-A (clone eBio17B7, #48717782, 1:100 dilution, eBioscience) (for Th1 and Th17, respectively).
To analyze the intracellular distribution of active RhoA, goat anti-GST antibodies (#27457701, 1:200, Merck) and FITC-labelled donkey anti goat secondary antibodies (#A11055, 1:500, Thermo Fischer Scientific) were used.
For western blotting, antibodies directed against P2Y10 (Life Technologies GmbH, #PA570914, 1:1000) or GAPDH (Cell Signaling Technology, #2118, 1:1000) as well as horseradish peroxidase-conjugated antibodies directed against Rabbit or Mouse IgG (1:3000, Cell Signaling Technology Europe) were used.
For immunohistochemical stainings, we used anti-Mac3 antibodies (clone M3/84, #550292, 1:200 also known as CD107b or LAMP-2; BD Pharmingen).
All antibodies were validated for the respective application by the vendor.

Validation

For flow cytometry, only standard antibodies were used. The P2Y10 antibody was validated using P2Y10-deficient cells (see Fig. 1c of the manuscript).

Eukaryotic cell lines

Policy information about cell lines

Cell line source(s)	COS-1 cells, obtained from ATCC.
Authentication	Fresh from ATCC; no further authentication.
Mycoplasma contamination	tested negative
Commonly misidentified lines (See ICLAC register)	We did not use commonly misidentifed cell lines

Animals and other organisms

Policy information about studies involving animals; ARRIVE guidelines recommended for reporting animal research

Laboratory animals	Mouse, C57BL6/J background (control and P2Y10-deficient), age 7-16 weeks, gender mixed.
Wild animals	not involved
Field-collected samples	not involved
Ethics oversight	Animal experiments were approved by the Institutional Animal Care and Use Committee of the Regierungspräsidium Darmstadt and in accord with Directive 2010/63/EU of the European Parliament on the protection of animals used for scientific purposes.

Note that full information on the approval of the study protocol must also be provided in the manuscript.

Human research participants

Policy information about studies involving human research participants

Population characteristics	PBMC from healthy donors (lab staff) and MS patients; Age 32-72 years, mixed male and female, for details, see manuscript.
Recruitment	Lab staff (healthy donors) and Department of Neurology, Frankfurt University (MS patients). Patients were scheduled for routine follow-up in the Neurology department.
Ethics oversight	Experiments with human samples were performed according to the regulations of the local ethics committee of the Hessian Regional Medical Board (Ethikkommission des Fachbereiches Medizin der Goethe-Universität Frankfurt; AZ 110/11), and informed consent was obtained from all subjects.

Note that full information on the approval of the study protocol must also be provided in the manuscript.

Flow Cytometry

Plots

Confirm that:

- [x] The axis labels state the marker and fluorochrome used (e.g. CD4-FITC).
- [x] The axis scales are clearly visible. Include numbers along axes only for bottom left plot of group (a 'group' is an analysis of identical markers).
- [ ] All plots are contour plots with outliers or pseudocolor plots.
- [x] A numerical value for number of cells or percentage (with statistics) is provided.

Methodology

Sample preparation	For the analysis of murine leukocyte populations, lymphatic organs were harvested, minced, filtered and stained with the indicated antibodies.<br>For the analysis of spinal cord infiltrating leukocytes, spinal cords were homogenized with a glass tissue homogenizer in PBS containing 1% glucose and 0.1% BSA. After centrifugation, spinal cords were resuspended in 6 ml of 30 % Percoll (Sigma-Aldrich) and layered on a gradient consisting of 4 ml 45 % Percoll and 2 ml 70 % Percoll. Gradients were spun for 20 minutes (970xg, room temperature, without break) and interphases between the layers harvested. After washing, cells were stained with fluorochrome-labelled antibodies and analyzed by flow cytometry.
Instrument	FACS Canto II
Software	FACS DIVA v6.1.2
Cell population abundance	not applicable
Gating strategy	The gating strategies are depicted in Supplemental Figure 11

- [x] Tick this box to confirm that a figure exemplifying the gating strategy is provided in the Supplementary Information.
